# One step excision combined with unilateral transforaminal intervertebral fusion *via* minimally invasive technique in the surgical treatment of spinal dumbbell-shaped tumors: A retrospective study with a minimum of 5 years’ follow-up

**DOI:** 10.3389/fsurg.2022.939505

**Published:** 2022-09-13

**Authors:** Jianfeng Pan, Yutong Gu, Feng Zhang

**Affiliations:** ^1^ Department of Orthopedic Surgery, Zhongshan Hospital Fudan University, Shanghai, China; ^2^ Department of Orthopedic Surgery, Shanghai Tenth People's Hospital, School of Medicine, Tongji University, Shanghai, China; ^3^ Shanghai Southwest Spine Surgery Center, Shanghai, China; ^4^ Joseph M. Still Burn and Reconstructive Center, Jackson, Mississippi, United States

**Keywords:** dumbbell-shaped tumors, minimally invasive technique, one step, unilateral transforaminal intervertebral fusion, traditional open surgery

## Abstract

**Introduction:**

Spinal dumbbell-shaped tumors are rare, usually benign tumors with intraspinal and paravertebral components connected through intervertebral foramen. Complete excision is often performed through traditional open surgery (TOS). The efficacy and long-term outcomes of minimally invasive surgery (MIS) have not been reported to date in resection of dumbbell-shaped spinal tumors.

**Purpose:**

The purpose was to evaluate the efficacy and long-term outcomes of minimally invasive resection combined with unilateral transforaminal intervertebral fusion (TIF) through comparing with TOS in the treatment of spinal dumbbell-shaped tumors.

**Methods:**

Fifteen patients underwent MIS and 18 patients underwent TOS. Thoracic dumbbell-shaped tumors were directly exposed after removal of costotransverse joints, adjacent rib components, unilateral hemilamina, and facet joints. Lumbar dumbbell-shaped tumors were completely exposed after removal of transverse processes, unilateral hemilamina, and facet joints. Whether for minimally invasive resection or traditional open removal, dumbbell-shaped tumors were completely excised and unilateral TIF was performed to guarantee spinal stability. All patients were followed up for 5 years at least.

**Results:**

The mean length of surgical incision for two groups was 3.47 ± 0.37 vs. 6.49 ± 0.39 cm (*p* < 0.05). The average duration of the operation was 131.67 ± 26.90 vs. 144.17 ± 23.59 min (*p* > 0.05). The mean blood loss was 172.00 ± 48.79 vs. 285.83 ± 99.31 ml (*p* < 0.05). No blood transfusions were required in the two groups. The median length of hospitalization was 6 vs. 10 days (range: 5–8 vs. 7–14 days). The patients of two groups were monitored for an average of 65.93 ± 3.88 vs. 65.78 ± 3.56 months. At 5-year follow-up, all patients presented with normal neurological function (American Spinal Injury Association scale E). The Oswestry Disability Index in the MIS group decreased significantly more than the TOS group. No spondylolisthesis or spinal instability were found in the follow-up period. There was no recurrence of any spinal tumor 5 years after surgery.

**Conclusions:**

Spinal dumbbell-shaped tumors can be safely and effectively treated with minimally invasive resection combined with unilateral TIF. Compared with TOS, MIS offers a reduced length of surgical incision, blood loss, hospital stay, and postoperative pain. This surgical protocol might provide an alternative for the treatment of spinal dumbbell-shaped tumors.

## Introduction

Spinal dumbbell-shaped tumors are rare lesions located inside and outside the dura or spinal canal. As the tumor portions are connected through the intervertebral foramen, the tumors resemble a dumbbell. The most common types of spinal dumbbell-shaped tumors are derived from the spinal nerve sheath. These include schwannomas, neurofibromas, and neurilemmomas (1, 2). Although majority are benign, they usually compress the nerve root and spinal cord and result in progressive pain or neurological deficits. Spinal dumbbell-shaped tumors most often occur in the cervical and thoracic regions, and lumbar tumors are relatively rare.

The treatment for spinal dumbbell-shaped tumors is gross total resection (GTR), which can alleviate clinical symptoms and relieve compression on neural structures. Traditionally, spinal tumors are surgically resected through open approaches such as posterior, posterolateral, combined posterior, and anterior approaches. Open surgical excision requires a large amount of paraspinal muscle displacement from bony components to clearly expose the tumors. These procedures are associated with significant potential complications (3). Recently, emphasis has been placed on minimally invasive techniques to reduce paraspinal tissue disruption and enhance recovery after surgery, while achieving the same clinical outcomes.

Minimally invasive techniques have been extensively used in a variety of spinal pathologies for decades. Compared with open procedures, minimally invasive techniques have been shown to minimize muscle and soft tissue dissection, decrease blood loss, decrease hospitalization costs, shorten hospital stay, and improve recovery time (4–6). Biomechanically, minimally invasive techniques also lead to less spinal destabilization than open surgeries (7, 8). Based on these advantages, minimally invasive techniques have been introduced to treat spinal tumors (9). In a previous paper, we reported two cases of thoracic dumbbell-shaped tumors treated with minimally invasive techniques (10).

To date, reports of treatment of spinal dumbbell-shaped tumors with minimally invasive techniques have been limited to case reports or small series (11, 12). The mid-term or long-term outcomes of minimally invasive resection through the paraspinal muscle approach combined with unilateral transforaminal intervertebral fusion (TIF) have rarely been reported following treatment of spinal dumbbell-shaped tumors. This study aimed to evaluate the efficacy and long-term outcomes of this minimally invasive technique by comparing with traditional open surgery (TOS) in resections of dumbbell-shaped spinal tumors.

## Patients and methods

### Patients

Approval for this study was obtained from the Medical Ethics Committee of Zhongshan Hospital. Before the procedure, informed consent was acquired from the patients. Between December 2013 and January 2015, patients who were diagnosed with spinal dumbbell-shaped tumors and underwent surgical resection combined with unilateral TIF were enrolled. The patients treated with minimally invasive surgery (MIS) were assigned to group 1. The patients treated with TOS were assigned to group 2. The localization of the dumbbell-shaped tumors and the distinction between benign and malignant tumors were determined by magnetic resonance imaging (MRI) scans.

### Surgical technique

All surgical manipulations were performed by the same senior surgeon (YG), who has over 25 years of experience in spine surgery. After being anesthetized, endotracheally intubated, and mechanically ventilated, the patient was turned prone on a radiolucent operating table. The correct area of the affected vertebrae was identified using anteroposterior and lateral fluoroscopy and Kirschner wires. The posterior surgical area was conventionally sterilized and draped. Preoperative computed tomography (CT) was performed to evaluate the pedicles of adjacent vertebrae and to determine the optimal entry angle and depth in the coronal and sagittal planes.

**Figure 1:**
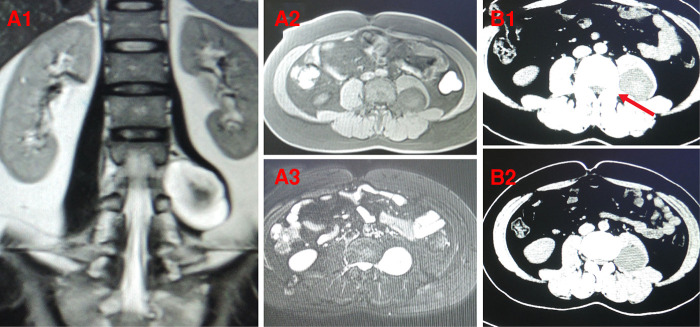
Preoperative MRI and CT showed lumbar dumbbell-shaped tumor of a 34-year-old male patient (case 5) (**A,B**). (**A1**) coronal MRI; (**A2**, **A3**) axial MRI; (**B1**, **B2**) axial CT. The pedicles of adjacent vertebra to lumbar dumbbell-shaped tumor (L3/L4) were intact. The red arrow indicates intact pedicle.

**Figure 2:**
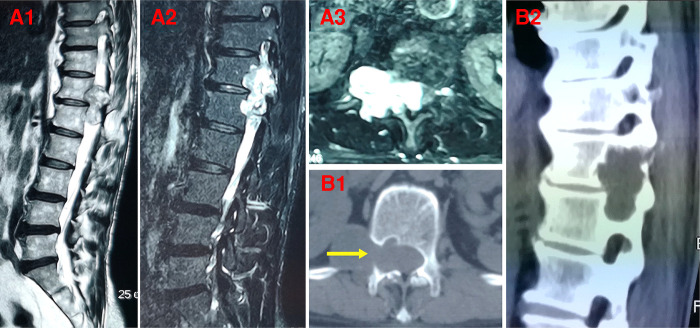
Preoperative MRI and CT showed thoracic dumbbell-shaped tumor of a 58-year-old male patient (case 15) (**A,B**). (**A1**, **A2**) sagittal MRI; (**A3**) axial MRI; (**B1**) axial CT; (**B2**) sagittal CT. The pedicles of adjacent vertebra to thoracic dumbbell-shaped tumor (T12/L1) were involved and damaged. The yellow arrow indicates the damaged pedicle.

When the pedicles of superior and inferior vertebrae adjacent to tumor were intact (Figures 1B1, B2), the tumor was completely resected from the lesion side, and unilateral TIF was performed on the ipsilateral side. For these patients, a paramedian mini incision was made 2 cm from the midline to access the dumbbell-shaped tumor and to insert the pedicle screws and cage. When the pedicles of superior and inferior vertebrae adjacent to tumor were damaged (Figure 2), the tumor was completely resected and the cage was inserted from the lesion side; unilateral pedicle screws fixation was performed on the contralateral side through a standard posterior midline incision about 35-mm and bilateral paraspinal muscle-splitting approaches.

**Figure 3:**
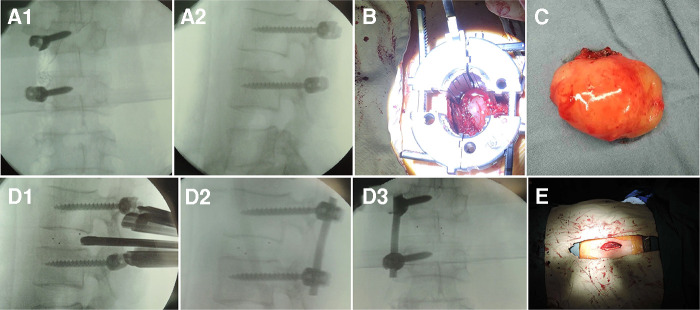
One-step excision combined with unilateral TIF *via* minimally invasive technique was performed to treat lumbar dumbbell-shaped tumors in case 5 as follows: (**A1**, **A2**) The pedicle screws were implanted into the vertebral body. (**B**) The dumbbell-shaped tumor was exposed through the expandable tubular retractor. (**C**) The dumbbell-shaped tumor was completely separated from iliopsoas muscle and excised in one step. (**D1**) The interbody cage was placed into the intervertebral interspace. (**D2**, **D3**) The rod was placed and fixed with two pedicle screws. (**E**) The paramedian mini incision was made 2 cm from the midline.

Blunt finger dissection between multifidus and longissimus muscles was performed to expose vertebral facet joints and transverse processes of the superior and inferior vertebrae. The pedicle screws were placed on the junction between the lateral facet wall and the superior third of the occurred transverse process. The cortical bone at the entry site to pedicle was decorticated and either a pedicle probe or a handheld curette was used to enter the pedicle. The continuity of the pedicle wall was confirmed using a small ball-tipped probe to ensure that there was no violation of the spinal canal or neuroforamen. The pedicle screws were implanted into the vertebral body, and anteroposterior and lateral fluoroscopy was performed to confirm the position of pedicle screws (Figures 3A1, A2).

**Figure 4:**
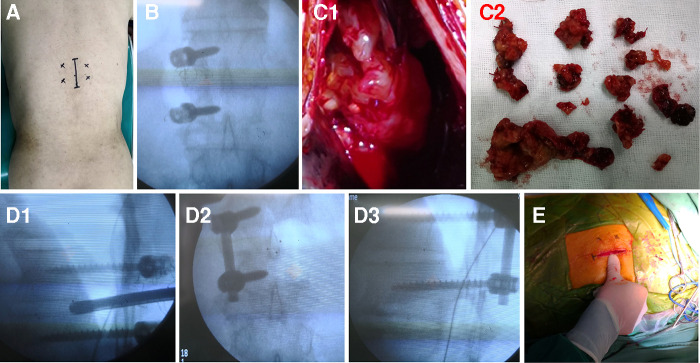
One-step excision combined with unilateral TIF *via* the minimally invasive technique was performed to treat thoracic dumbbell-shaped tumors with damaged adjacent pedicles in case 15 as follows: (**A**) the standard posterior midline incision about 35 mm and bilateral paraspinal muscle-splitting approaches were performed to place unilateral pedicle screws on the contralateral side and insert cage from the lesion side after the removal of dumbbell tumor. (**B**) The pedicle screws were implanted into the vertebral body. (**C1**, **C2**) The border of tumor is not clear and the tumor was excised in piecemeal fashion little by little. (**D1**) The interbody cage was placed in the intervertebral interspace. (**D2**, **D3**) The rod was placed and fixed with two pedicle screws. (**E**) The standard posterior mini incision was made from the midline.

Serial dilators were then used to create a muscle-splitting surgical channel into the target tumor area. An expandable tubular retractor was passed over the dilators to center over the tumor, and the retractor was fixed with a flexible arm to the operating table (Figure 3B). To completely expose the intraspinal component of dumbbell tumor, unilateral hemilaminectomy and total facetectomy were performed in piecemeal fashion using osteotomes and rongeurs. To completely expose the paravertebral component of dumbbell tumor, the costotransverse joints and adjacent ribs were removed for thoracic tumors while the transverse processes were removed for lumbar tumors. Then, the intercostal muscle or the intertransverse fascia was opened to access the tumor capsule. The paravertebral part of tumor could be separated from the thoracic pleura or iliopsoas muscle and completely pulled out using fingers if the tumor was easily mobilized (Figure 3C); otherwise, piecemeal excision of tumor was performed (Figures 4C1, C2). The nerve root involved was protected. After tumor resection, a standard ipsilateral discectomy was performed through the tubular retractor. The disc material and cartilaginous endplate were totally removed with the disc forceps and endplate scrapers. The interbody cage was filled with autograft bone and was placed into the intervertebral space (Figure 3D1). A rod was then placed and fixed with two pedicle screws after removal of the expandable retractor (Figures 3D2, D3). The wound was thoroughly irrigated, and a suction drain was inserted. For thoracic tumors, the thoracic pleura tears should be repaired, and placement of a chest tube was necessary depending on the hydrothorax or pneumothorax. The fascia was closed using absorbable sutures and the wound was closed in layers (Figure 3E).

No external braces were used after the operation. The patients were mobilized as soon as possible after surgery. After discharge, the patients were encouraged to resume their daily routines and were monitored as outpatients in the hospital ward.

### Clinical follow-up

All patients were assessed in terms of clinical outcomes on admission, after surgery, and at 3 months, 1 year, 2 years, and 5 years postoperatively. The pain intensity was assessed using a visual analog scale (VAS), and the motor/sensory outcomes were evaluated according to the American Spinal Injury Association (ASIA) scale. The Oswestry Disability Index (ODI) was performed preoperatively and 2 and 5 years after surgery. The length of surgical incision, intraoperative blood loss, operative time, and duration of hospitalization were analyzed. Spinal MRI scanning was performed before and after the operation to confirm the complete resection of tumor. X-ray and CT were performed on admission, after surgery, and 3 months, 1 year, 2 years, and 5 years after surgery. The fusion status was assessed according to the Bridwell posterior fusion grades (13). At the final follow-up, MRI was used in all patients to check if there was the recurrence of spinal tumor 5 years after surgery.

### Statistical analysis

All continuous variables were recorded and statistically analyzed by SPSS software (SPSS Inc., Chicago, IL). Values were expressed as mean ± SD. The level of significance was set at a *p*-value of ≤0.05.

## Results

Fifteen patients underwent minimally invasive resection and 18 underwent traditional open surgery. All tumors were radiographically benign. The tumors were located in the extradural region of the spinal canal and passed through intervertebral foramina to form paravertebral masses. For thoracic dumbbell-shaped tumors, costotransverse joints and adjacent rib components combined with vertebral laminae, and facet joints on the affected side were resected to expose the total tumor. For lumbar dumbbell-shaped tumors, ipsilateral transversectomy and hemilaminectomy combined with facetectomy were performed to remove the entire tumor. After GTR of dumbbell-shaped tumors in one step, all patients were concurrently treated with unilateral TIF to guarantee spinal stability. Before surgery, the ipsilateral pedicles of adjacent vertebra in one case of the thoracic tumors were involved as indicated by preoperative MRI and CT scans (Figure 2). For this patient, unilateral pedicle screw fixation was performed on the unaffected side. For other patients with intact pedicles, unilateral TIF was performed on the lesion side.

The characteristics of patients related to gender, age, involved level, and histopathological type are summarized in Tables 1 and 2. For the MIS group, there were eight men and seven women with a median age of 52 years. For the TOS group, there were ten men and eight women with a median age of 55 years. After surgery, they were monitored for at least 5 years. As shown in Table 3, the mean length of surgical incision for two groups was 3.47 ± 0.37 vs. 6.49 ± 0.39 cm (*p* < 0.05). The average duration of the operation was 131.67 ± 26.90 vs. 144.17 ± 23.59 min (*p* > 0.05), indicating that there was no significant difference. The mean blood loss was 172.00 ± 48.79 vs. 285.83 ± 99.31 ml (*p* < 0.05). No blood transfusions were required in the two groups. The median length of hospitalization was 6 vs. 10 days (range: 5–8 vs. 7–14 days). During the procedure, pleural disruption occurred in three cases of thoracic dumbbell-shaped tumors. In each of these cases, there was no obvious pneumothorax or hydrothorax on the x-ray immediately after surgery. Closed thoracic drainage was not performed.

**Table 1:** Results of patients performed *via* the minimally invasive technique.

Patient	Gender	Age	Involved level	Histopathological type	ASIA		
					Preop	3-month follow-up	5-year follow-up
1	M	34	L3/4	Ganglioneuromas	E	E	E
2	F	61	T10/11	Neurofibroma	E	E	E
3	M	68	T12/L1	Neurofibroma	E	E	E
4	F	46	T8/9	Ganglioneuromas	E	E	E
5	M	50	L3/4	Neurilemmoma	D	E	E
6	M	47	T7/8	Neurilemmoma	E	E	E
7	M	52	L1/2	Neurofibroma	E	E	E
8	M	18	T5/6	Shwannomas	C	E	E
9	F	54	L5/S1	Neurilemmoma	D	E	E
10	M	58	T12/L1	Neurilemmoma	E	E	E
11	F	57	T11/12	Shwannomas	D	E	E
12	M	62	T7/8	Shwannomas	E	E	E
13	F	49	L2/3	Neurilemmoma	D	E	E
14	F	45	T8/9	Shwannomas	E	E	E
15	F	55	T9/10	Neurofibroma	E	E	E

**Table 2:** Results of patients treated *via* traditional open surgical technique.

Patient	Gender	Age	Involved level	Histopathological type	ASIA		
					Preop	3-month follow-up	5-year follow-up
1	M	65	T7/8	Neurofibroma	E	E	E
2	M	62	T11/12	Neurilemmoma	E	E	E
3	F	55	T10/11	Ganglioneuromas	D	E	E
4	M	66	L3/4	Neurofibroma	E	E	E
5	F	57	T12/L1	Neurilemmoma	D	E	E
6	F	42	T8/9	Shwannomas	E	E	E
7	F	33	L1/2	Neurilemmoma	E	E	E
8	M	40	T9/10	Ganglioneuromas	D	E	E
9	F	27	L4/5	Neurofibroma	E	E	E
10	M	53	T11/12	Neurilemmoma	E	E	E
11	M	67	T9/10	Shwannomas	E	E	E
12	F	41	L1/2	Neurofibroma	E	E	E
13	M	58	T12/L1	Ganglioneuromas	E	E	E
14	M	53	T6/7	Shwannomas	D	E	E
15	F	47	L2/3	Neurilemmoma	E	E	E
16	M	55	T11/12	Neurofibroma	E	E	E
17	F	59	L1/2	Neurilemmoma	D	E	E
18	M	63	T10/11	Neurilemmoma	E	E	E

**Table 3:** Comparison of variables between two groups.

Variables	MIS	TOS	*p*-value
Operation time (min)	131.67 ± 26.90	144.17 ± 23.59	0.16
Blood loss (ml)	172.00 ± 48.79	285.83 ± 99.31	<0.05
Surgical incision (cm)	3.47 ± 0.37	6.49 ± 0.39	<0.05
Hospitalization (range/median, days)	5–8/6	7–14/10	<0.05
Follow-up period (months)	65.93 ± 3.88	65.78 ± 3.56	0.91

MIS, minimally invasive surgery; TOS, traditional open surgery.

Postoperative CT showed that the spinal dumbbell-shaped tumors were completely removed *via* the one-step minimally invasive technique. GTR was achieved in all patients. Histopathological analysis showed that the resected tumors were benign nerve sheath tumors (Tables 1, 2). The patients of two groups were monitored for an average of 65.93 ± 3.88 vs. 65.78 ± 3.56 months. There were no procedure-related complications. All patients returned to normal activities within 4 weeks.

During the follow-up, clinical outcomes were assessed by VAS, ODI, and ASIA. As for pain intensity in the symptomatic region of chief complaint, the preoperative VAS of two groups was 8.47 ± 1.06 vs. 7.89 ± 1.18, indicating that there was no significant difference (Table 4). After surgery and 3 months later, VAS score in the MIS group was lower than that in TOS group (*p* < 0.05). At 1-, 2-, and 5-year follow-up, there were no significant differences between MIS and TOS groups in the assessment of VAS score. As for ODI assessment (Table 5), the MIS group was higher than the TOS group before surgery, while at 2- and 5-year follow-up, the MIS group was lower than the TOS group (*p* < 0.05). This indicated that ODI in the MIS group decreased significantly more than the TOS group. As for neurological motor/sensory outcome, ASIA grade improved in all patients. In the MIS group, five patients had improvement of neurological function with ASIA scale to E after 3 months (four from D to E and one from C to E). The remaining 10 patients had normal neurological function (ASIA scale E) preoperatively and postoperatively. In the TOS group, five patients had improvement of neurological function with ASIA scale from D to E after 3 months. The remaining 13 patients had normal neurological function (ASIA scale E) preoperatively and postoperatively. At 5-year follow-up, all patients had normal neurological function (ASIA scale E).

**Table 4:** VAS pain assessment of the two groups.

Group	Preoperative	Postoperative	3 month	12 month	2 year	5 year
MIS	8.47 ± 1.06	2.07 ± 0.88	0.53 ± 0.52	0.27 ± 0.46	0.27 ± 0.46	0.27 ± 0.46
TOS	7.89 ± 1.18	3.83 ± 0.86	1.22 ± 0.81	0.67 ± 0.69	0.50 ± 0.62	0.50 ± 0.62
*p*-value	0.15	<0.05	<0.05	0.06	0.23	0.23

VAS, visual analog scale; MIS, minimally invasive surgery; TOS, traditional open surgery.

**Table 5:** ODI assessment of the two groups.

Group	Preoperative	2 year	5 year
MIS	79.73 ± 4.27	26.93 ± 6.09	21.20 ± 2.70
TOS	77.00 ± 2.93	35.00 ± 4.77	30.33 ± 3.65
*p*-value	<0.05	<0.05	<0.05

ODI, Oswestry Disability Index; MIS, minimally invasive surgery; TOS, traditional open surgery.

**Figure 5:**
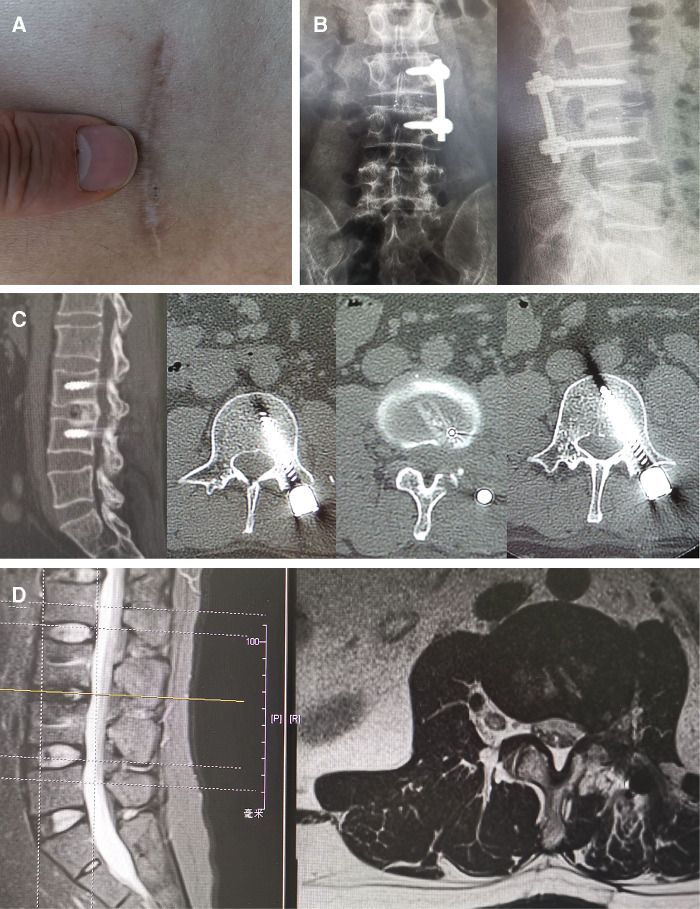
X-rays and CT scans showed the fusion of intervertebral segments was achieved, and there was no failure of internal fixation and occurrence of spinal deformity at 5 years after surgery. Grade I fusion was attained in a lumbar dumbbell-shaped tumor patient at 5-year follow-up. There was no recurrence of any spinal tumor 5 years after surgery confirmed by MRI examination. (**A**) The minimally invasive incision was shown 5 years after surgery. (**B**) x-rays; (**C**) CT scans. (**D**) MRI of lumbar dumbbell-shaped tumor patient (case 5).

**Figure 6:**
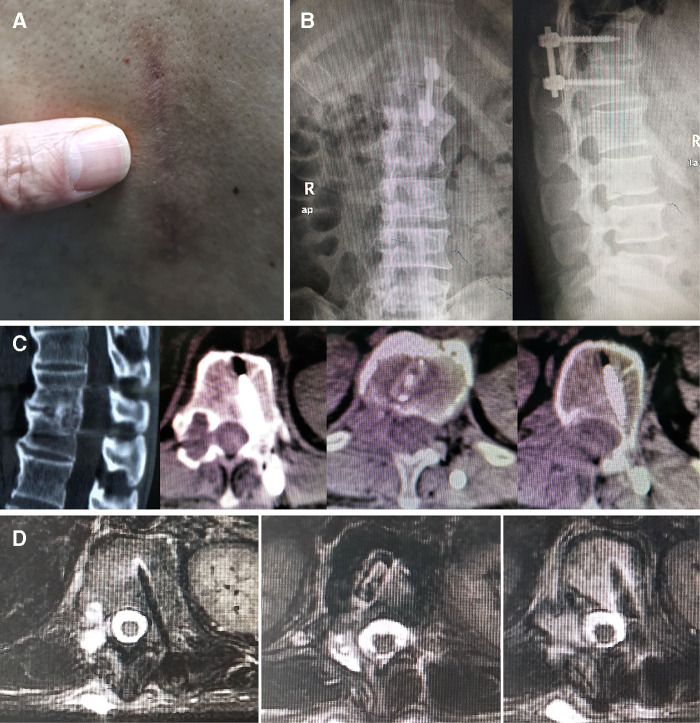
X-rays and CT scans showed the fusion of intervertebral segments was achieved, and there was no failure of internal fixation and occurrence of spinal deformity at 5 years after surgery. Grade I fusion was attained in a thoracic dumbbell-shaped tumor patient without fatigue of instrumentation at 5-year follow-up. There was no recurrence of any spinal tumor 5 years after surgery confirmed by MRI examination. (**A**) The minimally invasive incision was shown 5 years after surgery. (**B**) x-rays; (**C**) CT scans. (**D**) MRI of thoracic dumbbell-shaped tumor patient (case 15).

Postoperative x-rays and CT scans demonstrated that the pedicle screws and cages were properly positioned after surgery. During the follow-up period, there were no significant changes in the radiological examinations. No spondylolisthesis and spinal instability were found during the entire follow-up. Fusion of intervertebral segments was achieved in all patients after 2 years, including grade I in 11 segments (73.3%) and grade II in 4 segments (26.7%), based on the Bridwell grading system. No pedicle screw prolapses or rod failures were seen at the final follow-up. There was no recurrence of any spinal tumor 5 years after surgery confirmed by MRI examination (Figures 5, 6).

## Discussion

Minimally invasive approach has recently been used to treat spinal disorders to curtail the amount of soft tissue and bone removal. Successful minimally invasive approach of spinal surgery was described in our previous study and other studies regarding treatment of vertebral compression fractures and spinal degenerative pathologies (14–18). Potential advantages include decreased blood loss, lower hospitalization costs, less postoperative pain and narcotic use, shorter hospital stay, and quicker return to daily activities. These studies primarily focused on patients with vertebral compression fractures and degenerative pathologies. Minimally invasive strategies have been rarely reported in spinal dumbbell-shaped tumors. We reported our experience with minimally invasive resections of dumbbell-shaped spinal tumors combined with unilateral TIF.

TOS using open posterior midline approach typically necessitates a lengthy surgical incision. Moreover, it requires extensive dissection of paraspinal muscles from the underlying bony components. Bilateral laminectomies and radical ipsilateral facetectomy are usually performed to expose tumors completely. The open approach is associated with iatrogenic complications such as massive blood loss, sustained postoperative pain, potential wound infection, spinal instability, and deformities. Therefore, MIS for resection of spinal tumors was introduced to reduce approach-related iatrogenic complications. In this study, minimally invasive resection of dumbbell-shaped tumors, whose medial border was located in the extradural region of spinal canal and near the midline of vertebral canal, was performed using a unilateral paraspinal muscle approach with an expandable tubular retractor. Unlike open posterior midline approach, the minimally invasive approach preserves the supraspinous and interspinous ligaments, avoiding extensive stripping of paraspinal muscles from the bony components of the spine while providing adequate access to lamina, facet joints, and transverse processes. Thus, minimally invasive resection of spinal dumbbell-shaped tumors offers some advantages over traditional open resection (19). First, the MIS technique through the paraspinal muscle-splitting approach provides easy access to the dumbbell-shaped tumor after hemilaminectomy and facetectomy, allowing complete resection of tumors without any traction on nerve structures. This is beneficial to prevent postoperative neurological complications. Second, it preserves the ligamentous structures and the attachment of paraspinal muscles to bone, decreasing postoperative pain. Third, it reduces the operative blood loss and shortens the surgical incision and hospital stay. Finally, it facilitates to promote early postoperative rehabilitation of patients. In our study, MIS patients were able to mobilize postoperatively as soon as possible without additional external immobilization devices. However, MIS also presents the disadvantage of prolonged learning curves for surgeons.

These spinal dumbbell-shaped tumors pass through the intervertebral foramina to form paravertebral masses. There are two distinct components located in intraspinal and paravertebral compartments. The intraspinal portion of the tumor is located in the intracanal and intraforaminal region of the spinal canal, requiring ipsilateral hemilaminectomy or radical laminectomy combined with facetectomy on the affected side to expose and remove the tumor effectively (20, 21). The paravertebral portion of the tumor is located in extraforaminal region and may extend into the retropleural or retroperitoneal regions. For the paravertebral component extending into the retroperitoneal cavity, laparoscopy-assisted resection has been reported (22). For the paravertebral component extending into the posterior mediastinum, endoscopy-assisted thoracic surgery was performed to resect tumor pieces safely (23). Even for large retropleural components or for tumors in hard-to-reach locations, thoracoscopic surgery was effective for removal (24). In previous studies of thoracic dumbbell-shaped tumor treatment, the combination of thoracoscopic and posterior spinal surgery has been proven to be a successful alternative surgical procedure (25, 26). Nevertheless, there are various complications of thoracoscopic surgery, including pulmonary complication, intercostal neuralgias, shoulder girdle dysfunction, and chronic postoperative pain syndromes (27, 28). If spinal dumbbell-shaped tumors can be resected directly in one step, complications can be reduced. Payer et al. reported the excision of a dumbbell-shaped tumor using a single posterior midline approach with laminectomy and costotransversectomy (29). Rzyman et al. reported one-step removal of thoracic dumbbell-shaped tumors performed by the thoracic team alone through a posterolateral thoracotomy and extended foraminectomy (30). In our study, whether for thoracic or lumbar dumbbell-shaped tumors, the paravertebral component can be directly exposed and resected completely after removal of transverse processes or costotransverse joints and adjacent rib components in one step through minimally invasive approach. If the tumor border is clear, surgeons can use their fingers to separate and completely pull out the paravertebral part of the tumor; otherwise, piecemeal excision of the tumor could be performed. This technique avoids another transthoracic or retroperitoneal surgery to remove the paravertebral mass and reduces the rate of procedure-related complications.

Laminectomy and facetectomy usually result in spinal instability and deformity, which are of particular concern after multilevel laminectomy and facetectomy (31). Papagelopoulos et al. reported that spinal instability and deformity after multilevel laminectomy for resection of spinal tumors were not uncommon in children and young adults, and necessitated the fusion of intervertebral segments to correct postoperative deformity and stabilize the spine (32). In this study, unilateral hemilaminectomy and total facetectomy were performed to expose and resect dumbbell-shaped tumors. The biomechanical stability of the spine was largely destroyed because of the resection of the hemilamina and facet joint. Katsumi et al. reported that the occurrence of postoperative spinal deformity or instability was about 20% resulting from laminectomies for resection of spinal tumor (33). In another retrospective study by Wiedemayer et al., the occurrence of postoperative spinal deformity or instability was about 30% even when the laminar roof was reconstructed using titanium miniplates after laminectomies for resection of spinal tumor (34). In this study, with the aim of minimizing the occurrence of postoperative spinal instability and deformity, unilateral TIF was performed after resection of spinal dumbbell-shaped tumors. At the final follow-up, fusion of intervertebral segments was achieved in all patients, and there was no failure of internal fixation and occurrence of spinal deformity. This suggests that unilateral TIF provides sufficient mechanical support for spinal stability and prevents spinal deformity associated with postoperative axial back pain. Through the minimally invasive incision performed for resection, the pedicle screws and cages could be implanted for unilateral TIF without additional intraoperative disruption. Sometimes, even though the pedicles of superior and inferior vertebrae adjacent to tumor were damaged, unilateral pedicle screw fixation was performed on the contralateral side and the cage was inserted from the lesion side after the complete removal of tumor through a mini posterior midline incision and bilateral paraspinal muscle-splitting approaches.

Like all other surgical techniques, pedicle screw fixation is not without risk, as it can violate the spinal canal or neuroforamen to cause nerve injuries. In our study, the pedicles were located and probed in all four quadrants to ensure that a solid bone tube was present and no violation into the spinal canal or inferiorly into the neuroforamen occurred. During the follow-up, none of the patients were found to have any postoperative neurological complications. The postoperative x-ray and CT images showed that the pedicle screws and cages were properly positioned in all patients. The fusion of intervertebral segments was secure in all patients and no hardware failure was seen in any patient at the final follow-up. These findings suggest that this technique avoids procedure-related neurological deficits and guarantees safety of operation.

The efficacy of total resection and postoperative recovery is also of concern in the treatment of spinal dumbbell-shaped tumors. In this study, the dumbbell-shaped tumors in all patients were completely removed, and all patients showed improvement of neurological function after surgery in both MIS and TOS groups. For the minimally invasive technique, the operation-related variables (length of surgical incision, blood loss, hospital stay) and postoperative outcome in terms of pain improvement and procedure-related complications were superior to traditional open or other techniques (35).

## Conclusions

Minimally invasive resection through the paraspinal muscle approach combined with unilateral TIF in one step is a safe and effective surgical technique. Ipsilateral hemilaminectomy and facetectomy are sufficient to remove the intraspinal portion, and the paravertebral portion extending into the retropleural or retroperitoneal region can be concurrently removed after excising transverse processes or costotransverse joints and adjacent rib components. Following these procedures, unilateral TIF is performed to prevent postoperative spinal instability and deformity. If the pedicles adjacent to the tumor are not involved and intact, unilateral TIF is advocated on the lesion side. If the pedicles are involved, cages are inserted from the lesion side and unilateral pedicle screw fixation was performed on the contralateral side. Compared with traditional open technique, minimally invasive surgery offers a reduced length of surgical incision, blood loss, hospital stay, and postoperative pain.

## Data Availability

The original contributions presented in the study are included in the article/Supplementary Material, further inquiries can be directed to the corresponding author.
